# Positive *Pneumocystis jirovecii* Sputum PCR Results with Negative Bronchoscopic PCR Results in Suspected Pneumocystis Pneumonia

**DOI:** 10.1155/2018/6283935

**Published:** 2018-04-03

**Authors:** Kelly Pennington, John Wilson, Andrew H. Limper, Patricio Escalante

**Affiliations:** ^1^Division of Pulmonary and Critical Care Medicine, Department of Medicine, Mayo Clinic, Rochester, MN 55905, USA; ^2^Division of Infectious Diseases, Department of Medicine, Mayo Clinic, Rochester, MN 55905, USA

## Abstract

**Introduction:**

The diagnostic standard for *Pneumocystis jirovecii* pneumonia (PCP) is direct microscopic identification; however, in recent years, polymerase chain reaction (PCR) from bronchoalveolar lavage (BAL) samples to detect *Pneumocystis* nucleic acids has proven to be more sensitive and specific. Sputum samples have been presumed inferior to bronchoscopic samples secondary to variability and adequacy of sample collection. We observed several cases of positive sputum PCP-PCR results with negative PCP-PCR BAL results. The aim of the current study was to further characterize the clinical setting and outcomes in patients with positive sputum PCP-PCR samples and negative BAL PCP-PCR samples.

**Methods:**

We identified all patients who underwent *P. jirovecii*-PCR testing at Mayo Clinic between 2011 and 2016. Patients with a positive sputum and negative BAL sample collected within a 14-day time frame were identified and underwent further chart review for demographics, immunocompromised state, and clinical outcome.

**Results:**

From 2011 to 2016, 4431 respiratory samples from 3021 unique patients were tested for the presence of *P. jirovecii* by PCR. Fifty-five samples (1.2% of all samples collected) belonging to 24 unique patients (0.79% of patients tested) were identified as having a positive and negative sample collected within 14 days. Of these 24 patients, 10 (46%) patients had a positive sputum or tracheal secretion sample with negative BAL or bronchial washings. Out of these 10 patients, 8 were immunocompromised and 9 underwent treatment for PCP with 6 patients improving.

**Conclusion:**

Our results suggest that discordant *P. jirovecii*-PCR testing results from sputum and bronchoscopic specimens are an infrequent occurrence. Patients with positive *P. jirovecii*-PCR sputum/tracheal secretion samples and negative bronchoscopic samples appear to be clinically infected and respond to PCP treatment. Sputum *P. jirovecii*-PCR testing may be a viable alternative to invasive testing.

## 1. Introduction

Although the incidence of *Pneumocystis jirovecii* pneumonia (PCP) is decreasing secondary to prophylactic medications and combination antiretroviral therapy, PCP continues to be the one of the most common opportunistic infections in immunocompromised individuals with significant morbidity and mortality [1]. The diagnosis of PCP can be challenging owing to nonspecific symptoms, concurrent infection, and inability to culture the organism [2]. Direct microscopic identification of *P. jirovecii* from induced sputum, bronchoalveolar lavage (BAL), or lung biopsy is the gold standard for diagnosis; however, molecular methods have improved upon the sensitivity and specificity of direct microscopic visualization [3]. A variety of polymerase chain reaction (PCR) assays utilizing different targets, PCR platforms, patient populations, specimen sources, and assay types have been studied [3–7]. Real-time PCR has proven to have many advantages over other techniques as it uses a closed system limiting the possibility of contamination and offers the ability to set detection thresholds at levels that correlate more closely with true infection rather than airway colonization [3, 8]. Real-time PCR detecting *Pneumocystis* nucleic acids in BAL and sputum samples is more sensitive and specific for identifying *P. jirovecii* than microscopic identification [9–12]. When *P. jirovecii* is detected without evidence of disease, it is often referred to as airway colonization; however, subclinical infection can be a consideration as well. The prevalence of either airway colonization or subclinical infection is unclear and may reflect both the immunocompetence of the host and specific PCR testing platform utilized. Our laboratory utilizes a real-time PCR assay, which detects *Pjcdc2*, a single-copy gene, and utilizes threshold settings that are specifically designed to detect a level of organisms associated with PCP infection, rather than “colonization” [8].

We recently have noted several cases of positive *P. jirovecii*-PCR by induced sputum with negative *P. jirovecii*-PCR by BAL in patients who were clinically infected. The sensitivity of *P. jirovecii*-real-time PCR from BAL specimens is believed to be ≥95% with a low false positive rate [3, 8, 13], and sputum samples have a presumably lower sensitivity owing to variable collection techniques. Most centers do not perform *P. jirovecii*-PCR on sputum samples secondary to this theoretical assumption that diagnostic sensitivity is lower. However, if sputum samples could provide an equivalent or superior means of diagnosis, then patients who may already be in severe respiratory distress could be spared an invasive procedure that can worsen respiratory failure. In the current study, we examined clinical scenarios of patients with positive *P. jirovecii*-PCR sputum/tracheal secretion samples and negative *P. jirovecii*-PCR BAL/bronchial washing samples to further delineate when sputum/tracheal secretions can be used to clinically diagnose and treat PCP.

## 2. Materials and Methods

The study was reviewed and approved by the Mayo Clinic Institutional Review Board (IRB number 16-006823). We utilized an advanced cohort explorer (ACE) system to retrospectively identify in our medical records all patients with *P. jirovecii*-PCR testing results who were evaluated at the Mayo Clinic Rochester campus between January 2011 and August 2016. PCR detection of *P. jirovecii* was performed using the LightCycler™ 2.0 platform PCR as previously described [3]. This is a “closed system” without open tubes to minimize the potential for cross contamination. This approach uses real-time PCR to amplify a 166 bp region of the single-copy *cdc2* gene of *P. jirovecii*. Unlike some other published PCR assays that rely on nested PCR of multicopy genes, such as mitochondrial large subunit genes, our assay detects the single-copy *Pjcdc2* gene and uses a detection threshold that best correlates with clinical infection [8]. Furthermore, we did not detect *P. jirovecii* nucleic acid in BAL of over 100 consecutive nonimmunocompromised patients that were prospectively studied [8]. Hence, our PCR assay was specifically designed and validated to detect PCP infection and not colonization. The sensitivity and specificity of this real-time PCR assay are 100% and 96%, respectively. The analytical sensitivity of the method is 5.6 copies per mcL of positive plasmid control. The analytical sensitivity in spiked, pooled BAL specimens was found to be 56 targets per mcL using the positive control plasmid. The specificity of the PCR assay was determined by evaluating DNA extracted from pure cultures of a variety of bacteria and fungi.

Patients with a positive *P. jirovecii*-PCR test collected from induced sputum or tracheal secretions and negative *P. jirovecii*-PCR test from bronchoalveolar lavage or bronchial washings collected within a 14-day time frame were identified as discordant and underwent further chart review for patient demographics, immunocompromised state, reason for the immunocompromised state, radiographic findings, and clinical outcomes. The rate of clinical improvement with PCP treatment as perceived by the treating providers was estimated.

## 3. Results

**Figure 1:**
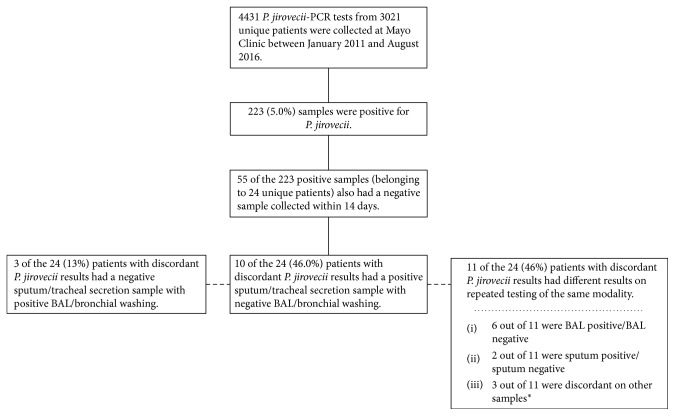
*P. Jirovecii-*PCR samples collected at Mayo Clinic from January 2011 to August 2016. PCR = polymerase chain reaction; BAL = bronchoalveolar lavage; ^∗^one patient had a total of 5 BAL PCP-PCR samples collected over a 28-day time period. The first sample was positive with the subsequent 4 samples collected from the same area reported as negative. Another patient with a positive PCP-PCR on lung biopsy and BAL had a subsequent negative PCP-PCR result on another lung biopsy sample from a different site. The third additional patient with discordant samples had a positive PCP-PCR collected on BAL from the right middle lobe and a positive PCP-PCR on the sputum sample; however, BAL from the right middle lobe collected 5 days later was negative.

We identified 4431 *P. jirovecii*-PCR testing samples that were collected from 3021 unique patients with 223 (5%) positive samples (Figure 1). Fifty-five samples (1.2% of all samples collected or 12.5% of all positive samples) belonging to 24 unique patients (0.79% of patients tested) were identified as having a positive and negative sample collected within 14 days. Of these 24 patients, 10 (46%) patients had a positive sputum or tracheal secretion sample with negative BAL or bronchial washings. Of note, only 3 of these 24 patients had a positive BAL/bronchial washing specimen with negative sputum/tracheal secretion.

Of the 10 patients with positive sputum/tracheal secretion *P. jirovecii*-PCR and negative BAL/bronchial washing *P. jirovecii*-PCR, 2 patients were not immunocompromised. The remaining patients were immunocompromised: renal transplant with subsequent posttransplant lymphoproliferative disease, metastatic neuroendocrine carcinoma on chemotherapeutics, multifocal pulmonary adenocarcinoma on chemotherapeutics, chronic lymphocytic leukemia, T-cell lymphoblastic lymphoma on chemotherapeutics, bone marrow transplant secondary to follicular lymphoma, bone marrow transplant secondary to multiple myeloma, and dermatomyositis on high-dose steroids (Table 1). While 6 of these patients qualified for PCP prophylaxis, only 2 patients were on PCP prophylaxis at the time of sample collection. Both of these patients were receiving trimethoprim-sulfamethoxazole.

One patient who was immunocompetent did not receive treatment for PCP and was diagnosed with small cell lung cancer as the precipitating event for his respiratory failure. Of the remaining 9 patients, 6 demonstrated clinical improvement with treatment directed at PCP and 3 died within 60 days of the initial *P. jirovecii*-PCR sample. Two of these patients died secondary to severe hypoxemic respiratory failure: one died within 24 hours of presentation and the other developed severe toxicity to trimethoprim-sulfamethoxazole resulting in renal failure and progressive pulmonary edema (per the patient's wishes, renal replacement therapy was not pursued). The third patient developed severe necrotizing pneumonia and died of septic shock.

The BAL sample was collected from an area with fewer radiographic infiltrates based on CT imaging in 2 of these 10 patients (i.e., one case with a sample collected from the lingula with perihilar-predominant infiltrates and another case with a sample collected from the right upper lobe with basilar-predominant infiltrates). Both of these patients improved with PCP treatment.

## 4. Discussion

Our results suggest that discordant *P. jirovecii*-PCR testing results from sputum and bronchoscopic specimens are an infrequent occurrence, but discordance among all positive specimens is not infrequent. Reasons for repeated PCP-PCR testing included pending PCP-PCR at time of second order (most common) and clinical deterioration despite appropriate treatment. In only two of the patients with positive sputum *P. jirovecii*-PCR and negative BAL *P. jirovecii*-PCR results, the reason for discordance was likely that the BAL was collected from an area with less dense pulmonary disease involvement on CT imaging. Both of these patients had focal areas of dense infiltrates (perihilar in one patient and basilar in the other patient), and it is possible that an optimal BAL sampling could have been associated with a better diagnostic yield in these cases. Both of these patients improved with PCP treatment, which also reflects a high clinical suspicion for PCP disease despite discordant testing results.

It is not entirely clear the cause of discordant testing results in the remaining patients with negative BAL and positive sputum *P. jirovecii*-PCR results. Potential explanations for this type of discordant testing results include the presence of an inhibitor of the PCR reaction and/or dilutional effects with BAL collection in patients with low PCP burden despite the high diagnostic sensitivity of BAL PCP real-time PCR testing [3]. Sample contamination can be a possible factor with any PCR technology and is another explanation for these discordant sample results; however, we minimized the risk of contamination by utilizing a closed PCR testing platform [8]. Additionally, most of these patients with discordant results were immunologically appropriate hosts for PCP, and many responded to antipneumocystis therapy.

The 3 patients who did not demonstrate clinical improvement with PCP therapy may have had PCP or *P. jirovecii* airway colonization with other coexistent processes causing significant pulmonary infiltrates and hypoxemia. One of these patients died within 24 hours of presentation making it difficult to determine the etiology of the respiratory failure. One of these patients was not immunocompromised and developed significant toxicities to trimethoprim-sulfamethoxazole. In this setting, it is difficult to determine whether the presenting respiratory process improved with PCP therapy. The last patient in this category developed necrotizing pneumonia and septic shock while on PCP therapy. It is likely that this patient had an alternative diagnosis leading to respiratory failure and death.

In conclusion, patients with positive *P. jirovecii*-PCR sputum/tracheal secretion samples and negative bronchoscopic samples appear to be clinically infected and respond to PCP treatment. Sputum *P. jirovecii*-PCR testing may be a viable alternative to invasive testing. This could be a more timely method for sample collection and would provide a safer alternative to bronchoscopic evaluation in patients who already have respiratory failure. Further studies comparing the sensitivity, specificity, and positive and negative predictive values for each sample type are needed.

## Figures and Tables

**Table 1:** Clinical characteristics of patients with positive sputum PCP-PCR testing and negative BAL/bronchial washing PCP-PCR results.

Reason for the immunocompromised state	Clinical course	Prior PCP prophylaxis	Response to PCP treatment	60-day mortality
None	New-onset mild respiratory failure later diagnosed with small cell lung cancer	None	Did not receive treatment	Living
History of renal transplant currently undergoing treatment for PTLD	Presented with dry cough and bilateral infiltrates on radiograph. No respiratory distress	None	Clinical improvement	Living
Metastatic neuroendocrine cancer on carboplatin and gemcitabine	Presented with significant respiratory failure (A-a gradient 350 mmHg) and bilateral upper lobe infiltrates	None	No clinical improvement	Died within 24 hours
None	Presented with cough and mild respiratory distress but progressed to overt respiratory failure requiring mechanical ventilation. Treatment initiated for PCP but developed thrombocytopenia and renal failure	None	No clinical improvement	Died within 2 weeks
Multifocal lung adenocarcinoma and steroid-dependent COPD	Presented with severe respiratory failure (A-a gradient 210 mmHg) requiring mechanical ventilation. Treated for PCP but continued to decline. Died secondary to sepsis from necrotizing pneumonia	Trimethoprim-sulfamethoxazole	No clinical improvement	Died within 2 weeks
Chronic lymphocytic leukemia with a remote history of alemtuzumab/rituximab	Presented with respiratory failure (A-a gradient 93 mmHg) and bilateral infiltrates on radiograph	None	Clinical improvement	Living
T-cell lymphoblastic lymphoma s/p 1 cycle of hyper-CVAD	Presented with hypoxic respiratory failure (A-a gradient 43 mmHg) and left mid/lower lung infiltrates. Improved with PCP treatment but was concurrently diagnosed with disseminated HSV and treated with acyclovir	None	Clinical improvement	Living
Follicular lymphoma s/p bone marrow transplant	Dry cough with evidence of pneumonitis on follow-up PET scan. No respiratory distress	None	Clinical improvement	Living
Multiple myeloma s/p bone marrow transplant	Presented with hypoxic respiratory failure and diffuse bilateral infiltrates on chest radiograph.	Trimethoprim-sulfamethoxazole	Clinical improvement	Living
Dermatomyositis on high-dose prednisone and methotrexate	Presented with severe hypoxic respiratory failure (A-a gradient 93 mmHg) and bilateral perihilar infiltrates on radiograph	None	Clinical improvement	Living

PCP = *Pneumocystis jirovecii* pneumonia; PCR = polymerase chain reaction; BAL = bronchoalveolar lavage; PTLD = posttransplant lymphoproliferative disorder; COPD = chronic obstructive pulmonary disease; s/p = status-post; hyper-CVAD = hyperfractionated cyclophosphamide, vincristine, doxorubicin, and dexamethasone.

## Data Availability

The data used to support the findings of this study are available from the corresponding author upon request.
